# The Robinson Remedy

**Published:** 1884-11

**Authors:** J. A. Robinson

**Affiliations:** Jackson, Mich.


### CORRESPONDENCE.

### "The Robinson Remedy."

JACKSON, MICH., Sept. 12, 1884.

EDITOR DENTAL REGISTER:

*Dear Sir:*—Your favor is at hand. In my paper before the American Dental Association, convened at Saratoga, I spoke of painless operations as a most desirable attainment to the profession. The remedy to this end I gave as *carbolized potash.* You will doubtless recollect that I said to you and Dr. Watling two years ago at Ann Arbor, "Mix equal parts of caustic potash with carbolic acid in a mortar and you will have a salifiable base that will coagulate the serum in the tubuli and of course cut off all communication by *coagulation;* and it will not injure bone tissue." This remedy will insure a painless operation while preparing a cavity, and in case of necessity may be applied twice. If the nerve is exposed the operation may be slightly painful at first, but an eschar will form so quickly and strongly that the capping with any of the oxyphosphates may proceed right along with more certainty of success than with any other preparation now in use or known. But this is not what I intended to say when I began this communication. I wish to tell you that the same preparation is the speediest and best cure for Pyorrhœa Alveolaris.

I have thoroughly tried the "Harlan Plan," viz: Peroxide of hydrogen and iodide of zinc, and I am certain that the *Robinson Remedy* is far better, more easily adapted, and does not take half the time. Try it!

Take some fibres of cotton and twist into a cord the size of fine yarn and long enough to envelope the tooth; wet the cord in the salifiable base solution, and carry it under the free margin of the gum, with a fine covered instrument about the size of a 70 needle at the point. The Harlan mode of preliminarily removing the calcarious deposit by pushing the chisel towards the apex of the teeth is excellent, for it scarifies the alveolar edge, allows the medicine to more readily take hold, and assists in destroying bacteria. I wrote to this effect to Dr. Stockwell a while ago, with compliments to Prof. Searl, of Springfield, Mass., with a request that Prof. Mayer give the chemical action of the medicine on the calcareous, or sanguinary deposit. I have not since heard from the suggestion made. I am now so old that I want this anodyne given as the "*Robinson Remedy*," so I can stay with the profession when I am gone. I don't believe any person of my ability has given the subject more thought or more attention since I have been interested in it, but there are a great many who *know more.* The pus under the free margin of the gum is annihilated on application of the medicament, as the astringent qualities of the preparation closes the pockets in a talismanic manner. But in preparing successive teeth, let the cotton remain round the necks of those first prepared, and do not prepare more than three at one sitting. You will herein find a "New Revelation," and not from any Island of Patmos, but from the Peninsula of Michigan.

You know I am somewhat enthusiastic, but I have tried this remedy in a good many cases with splendid results; in fact, it is *an absolute cure*—a miracle, so to speak. I desire you to try it, as you can find opportunity in almost every mouth. Of course *loose teeth* need to be wired in place till the pockets close.

The paper I read at Saratoga, Dr. Cushing told me he could not publish in part as it mentioned fibrous and metallic filling. I took the paper home with me but find on examination if I cut the *article*, it will destroy the harmony of the whole thing. Now while I wish to be loyal, I must say *I do hate red tape*, in that it seriously and alarmingly hinders the progress of the profession.

The mention of this remedy, in connection with Pyorrhœa Alveolaris, is not made in the Saratoga paper, but if you can use the recipe as given in any way to help those who are trying to progress you may do so.

With due sentiments of respect toward yourself, and an abiding wish for many years of prosperity to the DENTAL REGISTER, I remain Very sincerely your friend,

J. A. ROBINSON.

